# Surface Modification of Poly(ethylene-alt-tetrafluoroethylene) by Atmospheric Pressure Dielectric Barrier Discharge Plasma

**DOI:** 10.3390/polym17111519

**Published:** 2025-05-29

**Authors:** Xiaoshan Yan, Zuohui Ji, Xiaopeng Li, Yue Zhao, Zhen Li, Zhai Chen, Heguo Li

**Affiliations:** 1 State Key Laboratory of NBC Protection for Civilian, Institute of Chemical Defense, Beijing 100191, China; yanxiaoshan07@foxmail.com (X.Y.); lxpbuct@163.com (X.L.); sa11226532@mail.ustc.edu.cn (Y.Z.); m18811769090@163.com (Z.L.); chenzhai_jky@163.com (Z.C.); 2 Research Institute of Aerospace Special Materials and Processing Technology, Beijing 100074, China; zuohui1210@163.com

**Keywords:** ETFE membrane, DBD plasma, surface modification, hydrophilicity, peel strength

## Abstract

The fluororesin membrane emerges as an ideal chemical-protective clothing material due to its excellent permeation resistance. However, using a fluororesin membrane with a low surface energy for compounding fabrics is very challenging. Herein, we demonstrate a strategy to modify the surface of a poly(ethylene-alt-tetrafluoroethylene) (ETFE) membrane by the atmospheric pressure dielectric barrier discharge (DBD) of plasma under different working voltages, processing times, and concentrations of acrylic acid (AA) in a helium (He) atmosphere. The increase in the hydrophilicity of the ETFE membrane is confirmed by the wettability test, which shows a significant decrease in the water contact angle, from 96° to 50°, after plasma modification. The interfacial T-peel strength of an ETFE membrane composited with polyester fabric increased from 0.53 N/cm to 13.64 N/cm after plasma modification. Significantly, the T-peel strength of the composite using a modified ETFE membrane with ultrasonic washing could still reach 11.75 N/cm. Various characterization methods clearly disclosed the physical and chemical changes on the ETFE membrane surface, such as introducing the polar -COOH group at a nano-level, improving the roughness, decreasing the ratios of the F/C element, and increasing the ratios of the O/C element, suggesting using nano-level grafted polyacrylic acid (g-PAA) on the surface of the membrane by DBD.

## 1. Introduction

Chemical-protective clothing can provide effective protection for wearers who are exposed due to chemical warfare, industrial chemical production, chemical leakage accident disposal, and chemical anti-terrorism activities, preventing personnel from being attacked by toxic and hazardous chemicals in the forms of liquid, gas, or aerosol [1,2,3]. Generally, a single protective material has difficulty defending against all kinds of toxic and hazardous chemicals, and the design and fabrication of multilayered protective materials has been increasingly demanded. The fluororesin membrane has been recognized as a good candidate for protective materials, thanks to its excellent chemical penetration resistance, chemical corrosion resistance, mechanical properties, and flame-retardant properties [4]. The low surface energy of fluororesin membranes endows them with hydrophobic and oleophobic properties, which can make it difficult for toxin droplets to adhere to the membrane surface. On the other hand, however, the feature of low surface energy also increases the difficulty in integrating fluororesin membranes into other materials to obtain multilayered chemical-protective fabrics, which limits its applications in the field of chemical-protective clothing.

In order to make the integration of the fluororesin membrane easier, it is necessary to modify the surface of the membrane. Conventional surface modification methods include sodium naphthalene chemical treatment, irradiation grafting, plasma treatment, etc. [5,6,7,8]. The sodium naphthalene chemical treatment likely destroys the main structure of fluororesin, and the corrosive liquid can cause environmental pollution problems. Irradiation grafting features simple operation, a short processing time, and high efficiency, but it also requires expensive equipment and strict environmental conditions [9]. As a dry treatment method, plasma treatment has the ability to introduce diverse active functional groups to the treated surface in a short period and has the merits of low cost and high modification efficiency [10,11].

At present, nonthermal plasma modification of a polymer membrane surface is mainly divided into low-pressure plasma and atmospheric pressure plasma. In the process of low-pressure plasma, polymer membranes are first pretreated with plasma under a certain vacuum environment, and then, they interact with gases such as O_2_ to generate oxygen-containing radicals on the surface, which are used to initiate a grafting reaction [12,13]. Valerio et al. applied the low-pressure plasma technique to modify a polytetrafluoroethylene (PTFE) membrane and then immersed it in acrylic acid (AA) for graft polymerization [14]. They found that the surface roughness and the surface energy of the modified membrane were increased, but the surface-grafting state of the membrane was not well-demonstrated. Different from the low-pressure plasma strategy, atmospheric pressure plasma modification omitted the pretreatment step in a vacuum, which highly simplified the operation process and lowered the production cost, making it conducive to large-scale industrial production [15,16,17].

Nonthermal atmospheric pressure plasma is generally generated by the gas discharge method, including dielectric barrier discharge (DBD), corona discharge, glow discharge, etc. In particular, DBD has a barrier medium placed between two discharge electrodes, which can effectively prevent the generation of arc discharge and achieve a uniform and stable high-power and high-intensity discharge. At the same time, since the electrode can be covered with a barrier medium, it can effectively prevent the electrode from being corroded when dealing with corrosive gases. The wide range of applications of DBD plasma has received a lot of attention [18,19]. Though uniform atmospheric discharge is easily achieved in an inert gas atmosphere [20], the pure inert gas plasma-modified fluororesin membrane surface is unstable, making hydrophilicity, as well as other characteristics, easy to eliminate. Therefore, the introduction of other active groups to enhance the stability of the modified surface is necessary [21]. In general, polar and inexpensive AA is introduced into the atmospheric pressure plasma to improve the adhesion properties of the polymer membrane surface [20,22]. Pandiyaraj et al. [16,17,23] introduced AA into the modified atmosphere for plasma treatment at atmospheric pressure and investigated the change in hydrophilicity of the modified membrane surface. Hori et al. [24] investigated the bondable properties of the PTFE membrane modified by an atmospheric pressure plasma jet under an Ar/AA atmosphere. Chen et al. [25] applied plasma pretreatment on a PTFE membrane under an Ar atmosphere, followed by placing the membrane in an AA atmosphere and heating it with a water bath.

In this work, a fluororesin membrane poly(ethylene-alt-tetrafluoroethylene) (ETFE), with good chemical corrosion resistance and permeability resistance, was modified using atmospheric pressure DBD plasma with a high-voltage AC power supply, and the effects of the treatment conditions on the surface modification were studied, including the working voltage, treatment time, atmosphere composition and post-treatment ways. The plasma modification treatment was carried out under a pure He and AA/He atmosphere with varied concentrations, and modified membranes were ultrasonically washed with acetone to examine the stability of the modified surface. The surface morphology and chemical properties of the ETFE membranes before and after the modification were characterized by attenuated total reflectance–Fourier transform infrared (ATR-FTIR), infrared photo-induced force microscopy (IR PiFM), X-ray photoelectron spectroscopy (XPS), scanning electron microscopy (SEM), and atomic force microscopy (AFM), and the surface hydrophilicity and surface cohesiveness of the ETFE membranes before and after the plasma modification were tested.

## 2. Experimental Section

### 2.1. Materials and Chemicals

An ETFE membrane (EFC-0055(M)) from Daikin Industries in Shanghai, China with a thickness of about 50 μm was used in this work. Acrylic acid (CH_2_CHCOOH, AR), acetone (CH_3_COCH_3_, AR), sodium hydroxide (NaOH, AR), and potassium hydrogen phthalate (C_8_H_5_O_4_K, AR) were purchased from Sinopharm Chemical Reagent Co., Ltd in Beijing, China. All chemicals were used as received without further purification. Helium (>99.999%) was obtained from Beijing Haipu Gas Co., Ltd in Beijing, China.

### 2.2. Experimental Device

**Figure 1:**
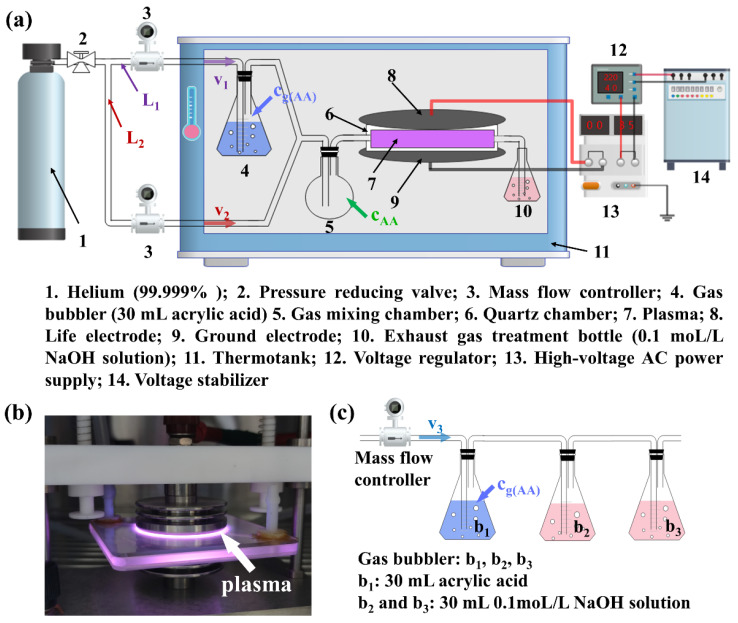
(**a**) Schematic diagram of the experimental setup, (**b**) discharge of DBD plasma device, (**c**) schematic diagram of cAA test.

The experimental setup is shown in Figure 1a, which mainly consists of the gas generation part and the atmospheric pressure DBD plasma discharge part. In the gas generation part, the flow rates, v_1_ and v_2_, of He in two gas pipelines, L_1_ and L_2_, are controlled by two mass flow controllers. The He in pipeline L_1_ passes through a gas bubbler containing 30 mL of AA, then carries the AA vapor to a chamber to be fully mixed with He from pipeline L_2_, and the mixed AA/He vapor finally enters the quartz chamber as the treatment atmosphere for the plasma modification process. In the atmospheric pressure DBD plasma discharge part, the working voltage of the high-voltage AC power supply (equipment model: CTP-2000K, Nanjing Suman Plasma Technology Co., Ltd. In Nanjing, China) is regulated by the voltage regulator, with a sine output voltage wave, a 4–18 kV peak voltage, and a frequency of 10.8 ± 0.1 kHz. The upper and lower aluminum electrode plates are connected to the high-voltage AC power supply with a quartz chamber sandwiched between the electrode plates as a barrier medium, constituting a DBD plasma generator. The thickness of the electrode plates is 2 mm, and the height of the internal gap is 4 mm, which can effectively avoid an electric arc and is beneficial to the generation of a uniform and stable plasma discharge. As shown in Figure 1b, the plasma discharge state is very uniform. To ensure a steady concentration of AA (*c_AA_*) during the experiment, both the gas generation part and the DBD plasma discharge part are placed in a thermotank with a constant temperature of 25 °C. The AA in the exhaust gas is absorbed after passing through an exhaust gas treatment bottle filled with 1 moL/L of NaOH solution. The *c_AA_* was calculated by using the acrylic acid generation concentration (c_gAA_) test device, as shown in Figure 1c, combined with the v_1_/(v_1_ + v_2_) ratio. The calculation process is described in the Supporting Information.

### 2.3. Plasma Modification of ETFE Membranes

ETFE membranes were ultrasonically washed for 30 min with acetone to remove the surface impurities, then dried in a vacuum. The achieved ETFE membrane was placed in a quartz chamber, and the atmospheric pressure DBD plasma surface modification was carried out under different atmospheres, with total gas flow rates (v_1_ + v_2_) of 100 mL/min or 200 mL/min, different working voltages for the high-voltage AC power supply, and different treatment times. During the experiment, the operation voltage ranged from 15 to 40 V to keep a stable discharge state under atmospheric pressure and ensure the safety of the experimental operation. In order to study the stability of the surface modification effect of ETFE membranes under AA/He, the partially modified ETFE membranes were ultrasonically washed in acetone for 15 min to remove the surface non-grafted polymers prior to the characterization and tests. The specific sample code names discussed in this work and the corresponding experimental conditions are shown in Tables S1 and S2 (Supporting Information). Taking the code name 1#-1-0%N as an example, “1#-1” indicates a treatment time of 5 s, a working voltage of 35 V, and a total gas flow of 100 mL/min, “0%” represents a v_1_/(v_1_ + v_2_) ratio of 0%, and “N” signifies that ultrasonic washing was not performed.

### 2.4. Characterization

The functionality of the membranes was characterized via Fourier transform infrared spectroscopy (FTIR, Frontier IR, PerkinElmer, in Waltham, MA, USA) using the attenuated total reflectance (ATR) mode. An atomic force microscope (AFM) with a vision–infrared–Raman coupled system (VistaScope, Molecular Vista Inc. in San Jose, CA, USA) based on the nano-infrared technology of photoinduced force microscopy (IR PiFM) was used to detect the nano-level infrared spectral characteristics of the ETFE membranes before and after modification. The IR PiFM is a new scanning probe spectroscopy technique to measure the interactions between the AFM probe and the samples in the non-contact mode, which can provide information on the chemical properties of the sample surface at the nanoscale [26]. The microscope is coupled to a Block QCL product with a wavenumber resolution of 0.5 cm^−1^ and a tuning range from 1900 to 800 cm^−1^ from Block Engineering.

The elemental composition and chemical states of the ETFE membrane are characterized via X-ray photoelectron spectroscopy (XPS, Thermo Scientific K-Alpha, in Waltham, MA, USA). The instrument parameters were an Al Kα (hν = 1486.6 eV) excitation source, a vacuum level of 5 × 10^−9^ mbar, and a pass energy of 150 eV. After calibrating the measurement data with the C1s peak (284.8 eV) binding energy, XPS Peak Fit (V4.1) software was used for peak fitting and data analysis. To investigate the depth of plasma modification, an argon gas cluster ion beam was applied for 0, 0.5, 1.0, and 1.5 min of etching, respectively. Subsequently, at each depth, the surface elemental composition was characterized using a PHI 5000 Versaprobe III (Ulvac-Phi, in Kanagawa, Japan), a multi-purpose scanning XPS microprobe. The analysis chamber’s vacuum level was below 4.78 × 10^−6^ Pa, and a monochromatic Al Kα X-ray source (Mono AlKa) was used with the analyzer in CAE scanning mode.

The surface of the ETFE membrane is coated with gold for 30 s prior to the scanning electron microscope (SEM, Gemini 300, in Baden-Württemberg, Germany) characterization. AFM (Dimension Icon, Bruker, in Billerica, MA, USA) is used to analyze the surface morphology of the ETFE membrane. The contact angle test (DSA100, Krüss GmbH, in Hamburg, Germany) was conducted to evaluate the hydrophilicity of ETFE membranes. A 5 μL deionized water droplet is placed on the membrane surface, and the water contact angle of the membrane surface is measured. Each group of samples is tested at 10 different locations to obtain the average water contact angle.

Polyester fabric is used as the substrate and is uniformly coated with a layer of adhesive (577H, Great Eastern Resins Industrial Co., Ltd., in Shanghi, China), which is subsequently bound with the ETFE membrane and dried at 60 °C for 12 h. The adhesive strength of the ETFE membrane is evaluated via a T-Peel test using a universal testing machine (34TM-5, Instron, in Boston University, Boston, MA, USA). The sample width is 25 mm, and the peeling speed is 100 mm/min [24]. At least five duplicated measurements were carried out for each sample.

## 3. Results and Discussion

### 3.1. ATR-FTIR and IR PiFM Analysis

**Figure 2:**
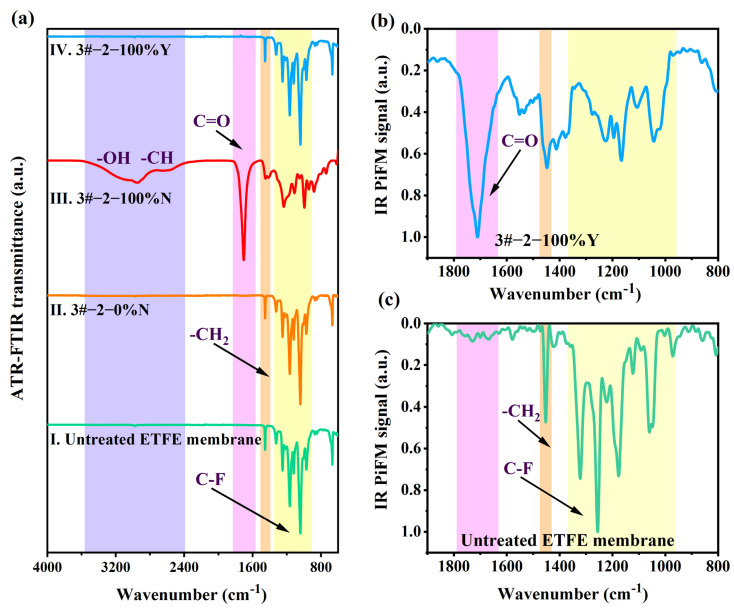
Surface chemistry of the ETFE membrane before and after plasma modification. (**a**) ATR-FTIR spectra comparing the untreated membrane and three modified membranes—3#-2-0%N, 3#-2-100%N, and 3#-2-100%Y. Specifically, in (II), ”3#-2-0%N” refers to the ETFE membrane treated with pure helium plasma; in (III), “3#-2-100%N” refers to the ETFE membrane treated with a helium/acrylic acid (C_g(AA)_ = 0.35 mmol/L) plasma; and in (IV), “3#-2-100%Y” refers to the ETFE membrane from (III) after ultrasonic cleaning. (**b**,**c**) IR PiFM spectra comparing 3#-2-100%Y to the untreated membrane.

**Scheme 1:**
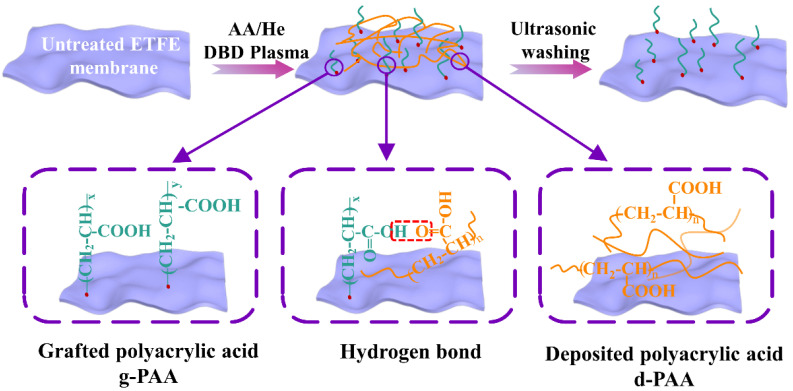
Changes in surface chemistry of ETFE membrane after AA/He plasma modification and acetone ultrasonic washing.

ATR-FTIR was used to characterize and analyze the functional groups of the ETFE membrane surface before and after plasma modification. Figure 2a shows the ATR-FTIR spectrum of the untreated membrane and the modified membranes (i.e., 3#-2-0%N, 3#-2-100%N, and 3#-2-100%Y). A series of strong peaks in the spectrum of the untreated ETFE membrane from 1350 cm^−1^ to 1000 cm^−1^ are assigned to the C-F of CF_2_ groups [27,28,29], and the sharp transmission peak at 1453 cm^−1^ is assigned to the C-H deformation vibration [30,31]. The infrared spectrum of the He plasma-modified 3#-2-0%N membrane was consistent with that of the untreated ETFE membrane, indicating that the modification by He plasma did not damage the main structure of the membrane. Differently, the spectrum of the 3#-2-100%N membrane shows remarkable changes compared with that of the untreated ETFE membrane. Specifically, there are a clear C=O stretching vibration peak at 1705 cm^−1^ [20,30] and a broad band at the range of 3700–2500 cm^−1^, which are attributed to the stretching vibration of -OH [32,33] and the stretching vibration of -CH [27], indicating that grafted polyacrylic acid (g-PAA) and deposited polyacrylic acid (d-PAA) have been introduced onto the surface of the ETFE membrane after AA/He plasma modification [13,15]. However, the characteristic peaks of C=O and -OH were absent in the infrared spectrum of the 3#-2-100%Y membrane after acetone ultrasonic washing, and the infrared spectrum became consistent with the untreated ETFE membrane. We hypothesize that, after the acetone ultrasonic washing, the d-PAA on the membrane surface was removed and only a trace of g-PAA remained on the surface, which was below the detection limit of ATR-FTIR. The proposed mechanism of surface modification by AA/He plasma and acetone ultrasonic washing is illustrated in Scheme 1. To confirm our hypothesis, the untreated membrane and 3#-2-100%Y membrane were further analyzed using IR PiFM at a nano-level depth. The IR PiFM technique analyzes the vibrational signal between the probe tip and the membrane surface [34,35,36]. Figure 2c shows the IR-PiFM spectrum of the untreated membrane, which also displays the C-H deformation vibration peak (1453 cm^−1^) and the C-F peaks (950–1400 cm^−1^), as mentioned in ATR-FTIR results. For the 3#-2-100%Y membrane, a sharp C=O characteristic peak at 1711 cm^−1^ was clearly observed in the IR-PiFM spectra (Figure 2b), confirming the presence of residual g-PAA on the membrane surface after the process of acetone ultrasonic washing [37].

### 3.2. XPS Analysis

**Figure 3:**
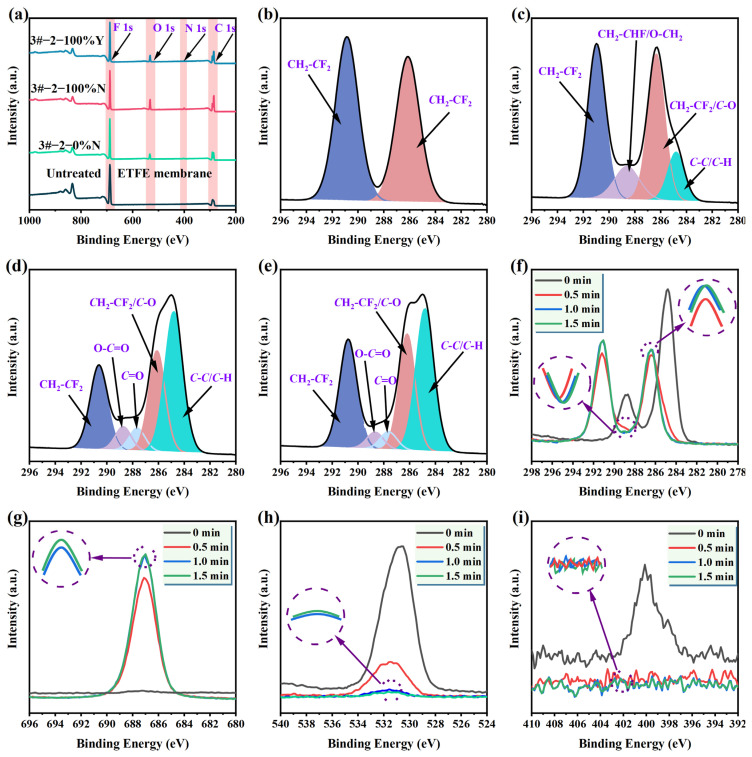
XPS spectra of ETFE membranes. (**a**) The XPS wide-scan spectra of the membrane surface. (**b**–**e**) The C 1s spectra of the untreated membrane and modified membranes 3#-2-0%N, 3#-2-100%Y, and 3#-2-100%N, respectively. (**f**–**i**) The C, F, O, and N etching spectra of the 3#-2-100%Y membrane, respectively. Specifically, “3#-2-0%N” refers to the ETFE membrane treated with pure helium plasma, “3#-2-100%N” refers to the ETFE membrane treated with a helium/acrylic acid (C_g (AA)_ = 0.35 mmol/L) plasma, and “3#-2-100%Y” refers to the ETFE membrane from (III) after ultrasonic cleaning.

The elemental composition and chemical state of the ETFE membrane surface before and after plasma modification were characterized by XPS. Figure 3a shows the XPS wide-scan spectra of the untreated ETFE membrane and modified membranes, i.e., 3#-2-0%N, 3#-2-100%N, and 3#-2-100%Y. It is observed that the peak intensities of the C and O elements of the modified membranes are higher than that of the untreated ETFE membrane, while the peak intensity of the F element is lower than that of the untreated ETFE membrane. These results indicate that the elemental concentration of the membrane surface has been changed by the plasma modification. The weak N peak in the spectra of the modified membranes should be attributed to the exposure of the membrane to air after plasma modification. Plasma modification introduces new polar groups to the ETFE membrane surface, resulting in changes in the elemental content of the membrane surface, as shown in Table S3. The F/C elemental ratio of the untreated ETFE membrane, 3#-2-0%N, 3#-2-100%Y, and 3#-2-100%N gradually decreased from 1.32, 0.85, and 0.74 to 0.47, respectively. In order to explore the mechanism of the changes in the F/C elemental ratio, the C 1s peaks of the four samples were fitted, with C representing the C elemental at the specific electron binding energy. Figure 3b shows the C 1s spectrum of the untreated ETFE membrane, consisting of CH_2_-CF_2_ at 286.1 eV and CH_2_-CF_2_ at 290.8 eV [4,38,39]. In comparison with the untreated membrane, the C 1s spectra of the 3#-2-0%N membrane (Figure 3c) shows two extra chemical states of the C bonds, the C-C/C-H peak at 284.8 eV [40], and the CH_2_-CHF/C-O peak at 288.6 eV [4,12], indicating that He plasma could generate C-F bond breakage on the membrane surface [41] and introduce an O element after contact with air [15,25]. Figure 3d,e show the C 1s spectra of the AA/He-modified 3#-2-100%Y and 3#-2-100%N membranes, which present the increased intensity of the C=O peak at 287.6 eV and the O-C=O peak at 288.6 eV compared with the 3#-2-0%N membrane. As shown in Table S3, the percentage of the CH_2_-CF_2_ peak area in the C 1s spectra for the untreated ETFE membrane, 3#-2-0%N, 3#-2-100%Y, and 3#-2-100%N decreases sequentially from 52.28%, 39.47%, and 23.27% to 27.50%, and the percentage of the C-C/C-H peak area increases from 0%, 12.05%, and 39.52% to 36.96%. For the 3#-2-0%N membrane, no AA was introduced during the plasma modification, which leads to fewer changes in the elemental content than that of the 3#-2-100%Y and 3#-2-100%N. The d-PAA on the surface of the 3#-2-100%Y was eliminated by ultrasonic washing with acetone, and only g-PAA remained. Meanwhile, both d-PAA and g-PAA were present on the surface of the 3#-2-100%N membrane. As a result, the C-C/C-H peak area ratio of 3#-2-100%Y was smaller than that of 3#-2-100%N, and the F/C ratio and the CH_2_-CF_2_ peak area ratio of 3#-2-100%Y were larger than those of 3#-2-100%N. In addition, a comparison of another two sets of data between 3#-1-60%N and 3#-2-60%N, 3#-1-60%Y and 3#-2-60%Y (Table S3) shows that the F/C element ratio of the membrane surface at a total flow rate of 200 mL/min is lower than that at a total flow rate of 100 mL/min, while the O/C element ratio at a total flow rate of 200 mL/min was higher than that at a total flow rate of 100 mL/min. When the c_AA_ concentrations were the same, the higher the flow rate, the more AA was passed through the quartz chamber per unit time, thus favoring the generation of g-PAA and d-PAA.

To investigate the thickness of the g-PAA layer, an Ar gas cluster ion beam was used to etch the 3#-2-100%Y membrane surface, and XPS spectra were recorded at 0 min, 0.5 min, 1.0 min, and 1.5 min of etching, respectively [42]. Here, 0 min refers to the state before etching. Figure 3f–i show the peak intensities of the C, F, O, and N elements on the 3#-2-100%Y membrane surface at different etching times. The C 1s spectrum shows the strong C-C/C-H peak at 284.8 eV and the relatively weak CH_2_-CF_2_ peak at 289 eV before etching. Then, the C 1s peaks shifted to the high electron binding energy after etching, and simultaneously, the intensity of the CH_2_-CF_2_ peak (290.8 eV) increased while that of the C-C/C-H peak (286.5 eV) declined along the etching process. With the increase in etching time, the intensity of the F peak in Figure 3g gradually increases while the intensity of the O peak in Figure 3h gradually decreases. These results reveal that the percentage of F element content on the membrane surface is smaller than that of the untreated membrane due to the introduction of AA, and the percentage of O element content is greater than that of the untreated membrane. However, with the deepening of the etching, the modification effect of AA/He plasma gradually became less evident, and the information regarding XPS spectra gradually became closer to that for the untreated ETFE membrane. The weak N peak at 0 min should originate from the air, and it almost disappeared after the etching, as shown in Figure 3i [40]. Collectively, these results show the plasma modification depth effect on the membrane surface, and after 1.5 min of etching, the relative content ratio of C, F, O, and N on the membrane surface tends to be similar to that of the untreated ETFE membrane. The results of XPS etching are consistent with the IR PiFM results, indicating that the depth of plasma modification on the ETFE membrane surface is at the nanometer level [30], and there is no damage to the main structure of the ETFE membrane [13].

### 3.3. Surface Morphology Analysis

**Figure 4:**
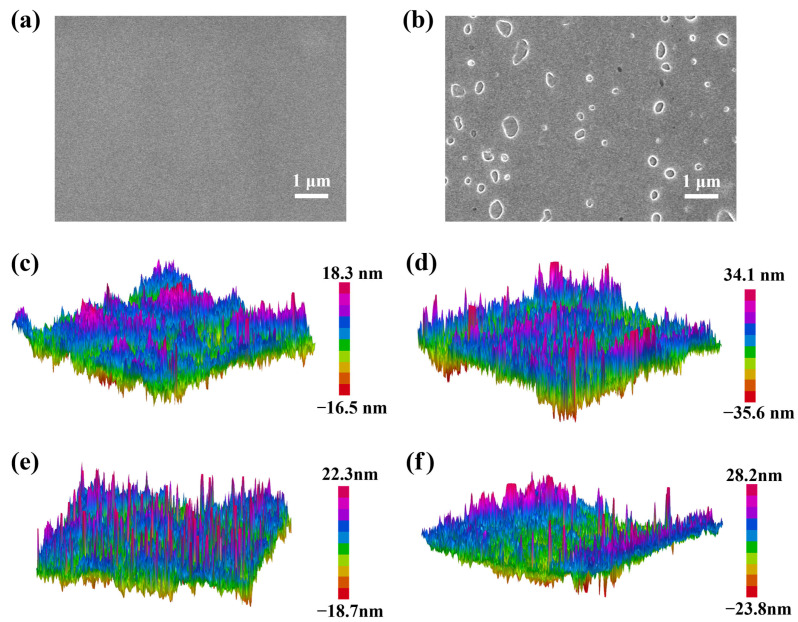
The SEM and AFM images of the membranes. SEM images of (**a**) the untreated ETFE membrane and (**b**) the 3#-2-100%N membrane. (**c**–**f**) AFM images of the untreated membrane, 3#-2-0%N, 3#-2-100%N, and 3#-2-100%Y, respectively. Specifically, “3#-2-0%N” refers to the ETFE membrane treated with pure helium plasma, “3#-2-100%N” refers to the ETFE membrane treated with a helium/acrylic acid (C_g (AA)_ = 0.35 mmol/L) plasma, and “3#-2-100%Y” refers to the ETFE membrane from (III) after ultrasonic cleaning.

SEM and AFM were used to observe the surface morphology of the ETFE membranes before and after plasma modification. The surface of the untreated ETFE membrane is smooth and uniform (Figure 4a), while the surface of the 3#-2-100%N modified by AA/He plasma presents several obvious polymer aggregates (Figure 4b) [24]. The roughness values Ra of the untreated membrane, 3#-2-0%N, 3#-2-100%N, and 3#-2-100%Y were determined to be 4.0 nm, 6.5 nm, 4.0 nm, and 5.6 nm, respectively [43]. In addition, the 3D AFM imaging also showed that the 3#-2-0%N membrane has the most obvious fluctuation on its surface, and the fluctuation degree of the surface morphology of 3#-2-100%Y, 3#-2-100%N, and the untreated ETFE membrane diminished in order (Figure 4c–f). In the 3#-2-0%N case, its surface roughness is larger than the untreated membrane, possibly due to the etching effect of He plasma [39]. In the case of AA/He plasma modification, the potholes etched by plasma might be filled with the accompanying PAA, including both d-PAA and g-PAA, making the overall surface roughness of the 3#-2-100%N membrane not as high as the 3#-2-0%N membrane but very close to the untreated membrane instead. It is reasonable to speculate that the etched potholes on the membrane surface can be exposed with the removal of PAA after the acetone ultrasonic-washing process. Therefore, the surface roughness of 3#-2-100%Y was observed to be larger than that of 3#-2-100%N.

### 3.4. Hydrophilicity of ETFE Membrane

**Figure 5:**
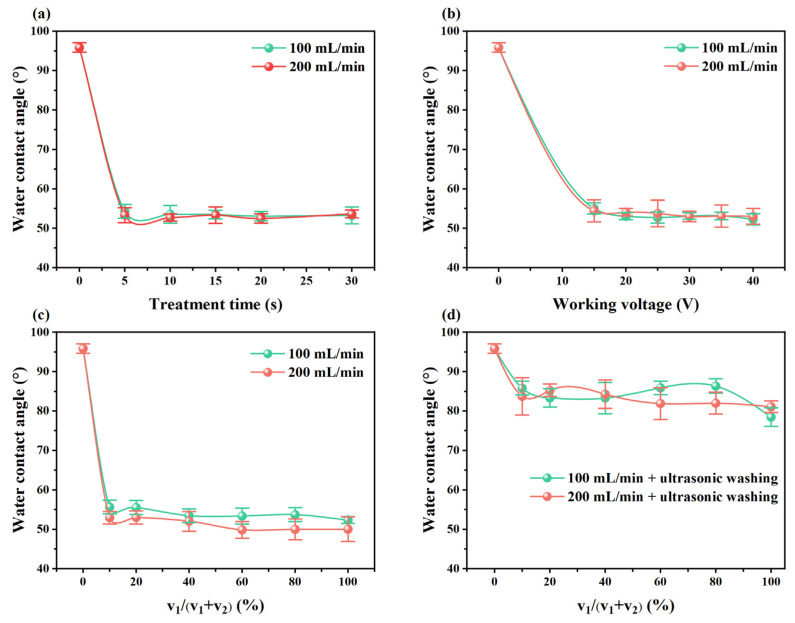
Effect of plasma modification conditions on the water contact angle, (**a**) treatment time, (**b**) voltage, (**c**) c_AA_, and (**d**) c_AA_ and ultrasonic washing with acetone. All zero points represent the untreated ETFE membrane.

Figure 5a–d, respectively, show the changes in water contact angle of the membrane surface after plasma modification under an He atmosphere and an AA/He atmosphere. As displayed in Figure 5a–c, the water contact angle of the modified ETFE membrane (96°~52°) decreased obviously compared with the untreated ETFE membrane (96°), indicating that plasma modification can significantly improve the hydrophilicity of the ETFE membrane surface. According to the previous study, some active sites can be generated after He plasma modification, which will be oxidized to active groups when exposed to air [39]. Thus, the introduction of active groups improves the hydrophilicity of the membrane [13]. It is generally believed that the increase in the treatment time provides the various active particles in the plasma with more opportunities to contact the membrane surface, and the increase in the working voltage endows these particles with higher energy. Thus, both will make the membrane surface produce more active sites, so that the contact angle shows a slightly decreasing trend with the extension of the treatment time and the increase of the working voltage. Considering the fluctuation of the experimental data and the safety of the experiment, a 15 s treatment time and a 35 V working voltage were chosen for the AA/He plasma modification. The introduction of g-PAA and d-PAA to the membrane surface via AA/He plasma further improves the hydrophilicity of the membrane surface, and the water contact angle decreased (96°~50°) with the increasing c_AA_ (Figure 5c). Ultrasonic washing with acetone can effectively remove the d-PAA from the membrane surface, reducing the content of polar groups and, thus, weakening the surface hydrophilicity of the membrane. As shown in Figure 5d, the contact angle of the membranes after acetone washing experienced a rebounding phenomenon, reaching 81°. The contact angle rebounding phenomenon is also supported by the results of AFM morphology analysis mentioned above because the g-PAA and d-PAA on the surface may affect the He plasma etching on the surface of the membrane. The small decrease in contact angle is basically caused by the hydrophilicity of the g-PAA, and the effect of plasma etching can be ignored.

### 3.5. Effect of Adhesion Strength of ETFE Membrane

**Figure 6:**
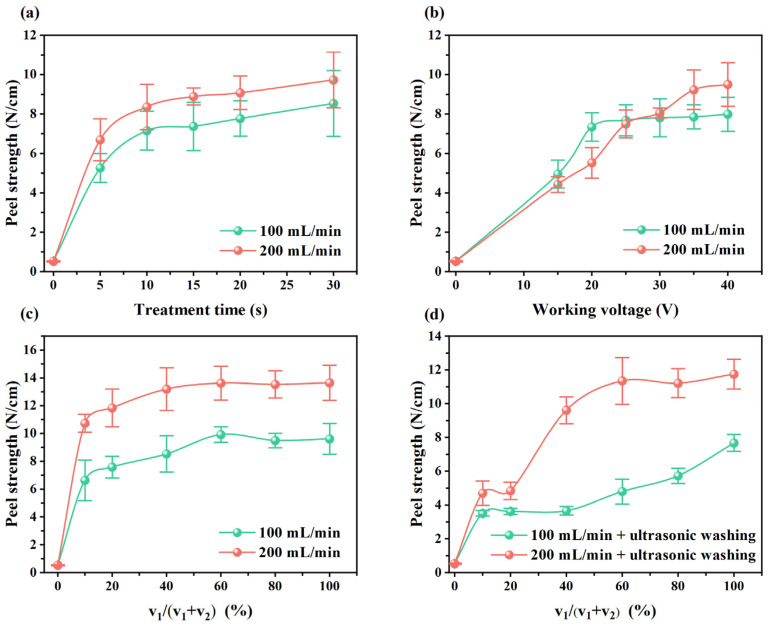
Effect of different plasma modification conditions on the peel strength, (**a**) treatment time, (**b**) voltage, (**c**) c_AA_, and (**d**) c_AA_ with ultrasonic washing. All zero points represent the untreated ETFE membrane.

As shown in Figure 6, the surface T-peel strength of the ETFE membrane modified by plasma was significantly improved. Compared with the surface morphology and water contact angle test results, the T-peel strength test results are greatly influenced by the experimental conditions. As discussed above, with the increase of working voltage and treatment time, the surface roughness enlarges, and the surface concentrations of active groups increase, which are conducive to the improvement of adhesion strength. Continuing to increase the working voltage and treatment time, the T-peel strength does not increase too much and gradually levels off. The T-peel strength increases from 0.53 N/cm for the untreated membrane to a maximum value of 9.72 N/cm for the 5#-2-0%N membrane with He plasma modification, which is 18.33 times that of the untreated membrane (Figure 6a,b). Within the experimental range, the peel strength gradually increases as the working voltage rises and the treatment time extends. This is because the weak boundary layer on the film surface gradually transitions into the material bulk, leading to a steady increase in bonding strength between the boundary layer and the bulk and, thus, an increase in peel strength. The gas flow rate also has a significant impact on peel strength. A higher flow rate delivers more active species to the discharge chamber per unit of time, improving the modification effect. However, when the helium plasma treatment reaches a certain level, the film’s surface morphology and chemical properties reach a dynamic equilibrium, and further increases in working voltage and treatment time lead only to a gradual plateau in peel strength, with no further notable increase. Compared with He plasma modification, AA/He plasma modification will additionally introduce AA to the surface of the membrane, which is beneficial to increase the number of polar groups on the membrane surface. Moreover, the g-PAA chain segments on the membrane surface also form a mechanical interaction with the adhesive chains to further improve the peel strength of the membrane [44,45,46,47]. In Figure 6c, the T-peel strength increases and then levels off along with the increase in c_AA_. In the low c_AA_ range, the increase in c_AA_ is favorable for the grafting of AA onto the membrane surface, and thus, the peel strength gradually increases with the increasing c_AA_. In the high c_AA_ range, the continued increase in c_AA_ makes the chance of AA monomers colliding increase, which thus facilitates the self-polymerization and deposition of the AA monomers on the membrane surface while the peel strength does not continue to increase. As shown in Figure 6c, the peel strength increases to 13.64 N/cm for the 3#-2-100%N membrane, which is about 25.74 times that of the untreated membrane. In Figure 6d, after ultrasonic washing with acetone, the peel strength is lower than before washing. Owing to the g-PAA residual on the surface of the after-washing membrane, the peel strength is still significantly improved compared with that of the untreated membrane, e.g., 11.75 N/cm for 3#-2-100%Y, which is about 22.17 times that of the untreated membrane. Under the same c_AA_ condition, the rapid gas flow rate is conducive for generating more d-PAA and g-PAA, and thus, a large difference in T-peel strength appears between the total flow rates of 100 mL/min and 200 mL/min, as shown in Figure 6c,d.

**Figure 7:**
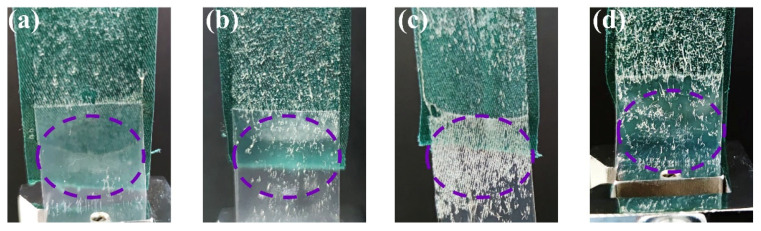
Photographs of bond damage during T peel testing of ETFE membrane composites. (**a**) Untreated membrane, (**b**) 3#-2-0%N membrane, (**c**) 3#-2-100%N membrane, and (**d**) 3#-2-100%Y membrane. Specifically, “3#-2-0%N” refers to the ETFE membrane treated with pure helium plasma, “3#-2-100%N” refers to the ETFE membrane treated with a helium/acrylic acid (C_g (AA)_ = 0.35 mmol/L) plasma, and “3#-2-100%Y” refers to the ETFE membrane from (III) after ultrasonic cleaning.

In addition, when the surface-bonding failure occurs, the adhesive residual on the membrane surface can reflect the peel strength of the membrane surface. The more adhesive residual left on the membrane surface, the better the peel strength of the membrane. As shown in Figure 7, the adhesive residual on the 3#-2-100%N surface is the highest, and those on 3#-2-100%N, 3#-2-0%N, and the untreated membrane decrease in sequence. These phenomena are consistent with the results of the T-peel strength test.

## 4. Conclusions

In this study, the surfaces of ETFE membranes were modified by atmospheric pressure DBD plasma under an He or AA/He mixed atmosphere, and the modification conditions, including working voltage, treatment time, and c_AA_, were investigated. The chemical properties and physical morphology of the ETFE membranes were characterized by ATR-FTIR, IR PiFM, and XPS. The results of XPS analysis demonstrated that plasma modification can significantly alter the elemental composition of the membrane surface and introduce numerous polar groups to the surface. The results of the IR PiFM demonstrated that the plasma-modified ETFE membrane under an AA/He atmosphere possessed a stable presence of g-PAA at the nano-layer level on the membrane surface, even after ultrasonic washing with acetone. AFM results showed that the surface roughness of the ETFE membrane increased significantly after being modified. Additionally, the modified membrane surface has good hydrophilicity, as the water contact angle of the ETFE membrane reduced from 96° to 50° after modification. The bonding test showed that the T-peel strength of the membrane material improved from 0.53 N/cm before modification to 13.64 N/cm after modification. The T-peel strength of the membrane after ultrasonic washing could still reach 11.75 N/cm. In this work, we provide a strategy to modify the ETFE membrane surface at the nanoscale in one step through atmospheric pressure DBD plasma under an AA/He atmosphere, which shows a good potential for industrial applications.

## Data Availability

The original contributions presented in this study are included in the article/Supplementary Materials. Further inquiries can be directed to the corresponding author.

## Supplementary Materials

The following supporting information can be downloaded at https://www.mdpi.com/article/10.3390/polym17111519/s1: Figure S1: At 25 °C, (a) c_g(AA)_ at different flow rates and (b) c_AA_ at different flow rates; Table S1: He plasma modification experimental conditions parameters; Table S2. AA/He plasma modification experimental conditions parameters; Table S3. Elemental composition, elemental ratio, and chemical bond concentration of ETFE membrane surface.
