# Supplementary material for: Surface Modification of Poly(ethylene-alt-tetrafluoroethylene) by Atmospheric Pressure Dielectric Barrier Discharge Plasma
Source: Polymers (Basel). 2025 May 29;17(11):1519. doi: 10.3390/polym17111519 (PMC12158105; doi:10.3390/polym17111519)
Supplement: Supplementary file 1 [file polymers-17-01519-s001.zip › polymers-3517104-supplementary.pdf]

Surface modification of poly(ethylene-alt-tetrafluoroethylene) by atmospheric pressure dielectric barrier discharge plasma

Xiaoshan Yan [1†], Zuohui Ji [2†], Xiaopeng Li [1], Yue Zhao [1], Zhen Li [1], Zhai Chen [1], Heguo Li [1,*]

[1] State Key Laboratory of NBC Protection for Civilian, Institute of Chemical Defense, Beijing 100191, China; yanxiaoshan07@foxmail.com

[2] Aerospace Institute of Advanced Materials & Processing Technology, Beijing, China; zuohui1210@163.com

* Correspondence: liheguo1972@126.com

† These authors contributed equally to this work.

Test of c_{AA}

In order to obtain the accurate value of c_{AA} during the modification process, a test experiment of AA generating concentration ($c_{g\ (AA)}$) are designed and carried out as shown in Fig. 1c. The b1 contains 30 ml AA, b2 and b3 are both 30 ml 0.1 moL/L sodium hydroxide solution ($n_{0(NaOH)}$ = 3 mmol). The He flow rate v3 is adjusted with a mass flow controller, and the He enters the b1 to generate an AA/He mixture, which is then passed into the b_2 and b_3 in turn. Each set of tests lasted 10 min (t = 10min). The AA in the AA/He mixture reacts with NaOH solution and consume a part of NaOH, as Eq. (1). The remaining NaOH ($n_{e\ (NaOH)}$) is acid-base titrated with 0.1 moL/L potassium hydrogen phthalate solution($c_{(C_8H_5O_4K)}$), as Eq. (2).

$$H_2C{=}CH{-}COOH + NaOH = H_2C{=}CH{-}COONa + H_2O \qquad (1)$$

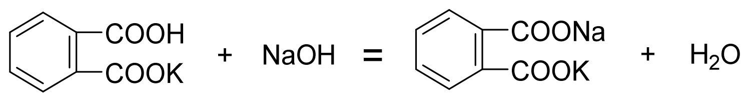

(2)

The consumed volume of potassium hydrogen phthalate solution $v_{x(C_8H_5O_4K)}$ is recorded, according to the equation:

$$c_{g(AA)} = \frac{n_{AA}}{v_1 t} = \frac{n_{x(NaOH)}}{v_1 t} = \frac{n_{0(NaOH)} - n_{e(NaOH)}}{v_1 t} = \frac{n_{0(NaOH)} - c_{(C_8H_5O_4K)} \times v_{x(C_8H_5O_4K)}}{v_1 t} \qquad (3)$$

calculating the consumed amount of NaOH substance, thereby obtaining the $c_{g(AA)}$. In order to verify whether the AA in the AA/He mixture can be completely absorbed by the NaOH solution in b_2, the same titration test method is used to titrate the NaOH solution in b_3. It is found that $n_{e(NaOH)} = n_{0(NaOH)}$ = 3 mmol, indicating that the gas is no longer contains AA when it enters b_3. It can indicate that the

NaOH solution in b_2 can completely absorb the AA in the AA/He mixture, ensuring the accuracy of the calculated $c_{g(AA)}$ results. After calculating $c_{g(AA)}$, the c_{AA} in the gas mixing chamber in Fig. 1a can be calculated according to equation 4:

$$c_{AA}=c_{g(AA)} \times \frac{v_1}{v_1+v_2} \qquad (4)$$

c_{AA} at different flow rates

The c_{AA} is tested at a constant temperature of 25°C, with v_1 in the range of 20 mL/min to 220mL/min. The test results found that $c_{g(AA)}$ at different flow rates within the experimental range is around 0.35 mmoL/L, as shown in Fig. S1a. In the Clausius-Clapeyron equation, the saturated vapor pressure is a function of temperature and is independent of the gas flow rate, so $c_{g(AA)}$ is a constant value [1-3]. According to the measured $c_{g(AA)}$, the c_{AA} can be calculated for the gas mixing chamber in Fig. 1a at different flow ratios. For example, when $v_1/(v_1+v_2)=0$, the modified atmosphere is pure He with $c_{AA}=0$. When $v_1/(v_1+v_2) = 60\%$, the c_{AA} in the modified is 0.21 mmoL/L. When $v_2 = 0$ and $v_1/(v_1+v_2) =100\%$, the maximum value of c_{AA} in the modified atmosphere is 0.35 mmoL/L. As shown in Fig. S1b, the c_{AA} in the modified atmosphere can be continuously adjusted from 0 to 0.35 mmoL/L by changing the value of $v_1/(v_1+v_2)$.

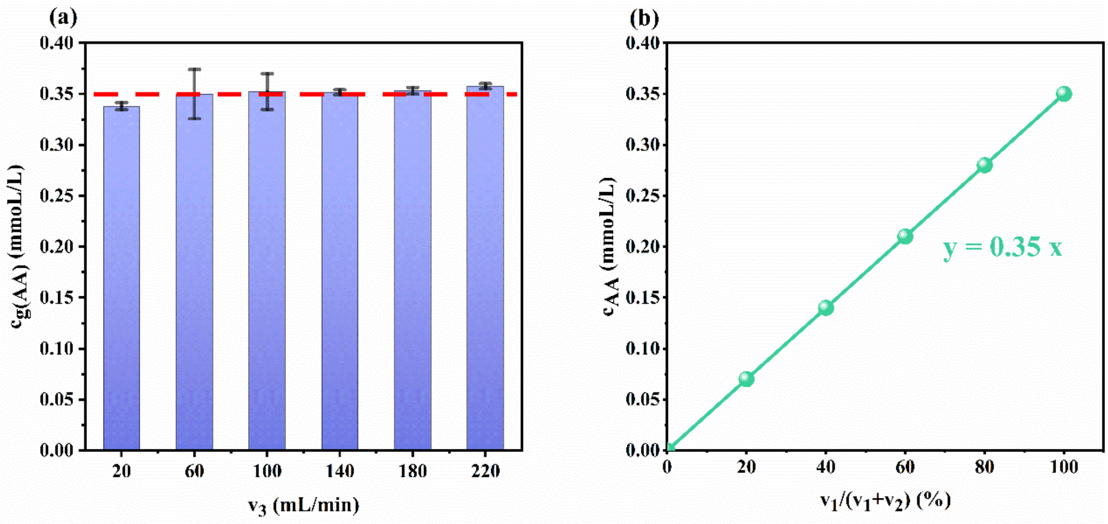

Fig. S1. At 25°C, (a) $c_{g(AA)}$ at different flow rates and (b) c_{AA} at different flow rates.

Table S1. He plasma modification experimental conditions parameters

No.	Treatment time (s)	Working voltage (V)	v_1+v_2 (mL/min)	$v_1/(v_1+v_2)$ (%)	ultrasonic washing (Yes/No)	Code Name
1	5	35	100	0	N	1#-1-0%N
2	5	35	200	0	N	1#-2-0%N
3	10	35	100	0	N	2#-1-0%N
4	10	35	200	0	N	2#-2-0%N
5	15	35	100	0	N	3#-1-0%N
6	15	35	200	0	N	3#-2-0%N
7	20	35	100	0	N	4#-1-0%N
8	20	35	200	0	N	4#-2-0%N
9	30	35	100	0	N	5#-1-0%N
10	30	35	200	0	N	5#-2-0%N
11	15	15	100	0	N	6#-1-0%N
12	15	15	200	0	N	6#-2-0%N
13	15	20	100	0	N	7#-1-0%N
14	15	20	200	0	N	7#-2-0%N
15	15	25	100	0	N	8#-1-0%N
16	15	25	200	0	N	8#-2-0%N
17	15	30	100	0	N	9#-1-0%N
18	15	30	200	0	N	9#-2-0%N
19	15	40	100	0	N	10#-1-0%N
20	15	40	200	0	N	10#-2-0%N

Table S2. AA/He plasma modification experimental conditions parameters

No.	Treatment time (s)	Working voltage (V)	v_1+v_2 (mL/min)	$v_1/(v_1+v_2)$ (%)	ultrasonic washing (Yes/No)	Code Name
21	15	35	100	10%	Y	3#-1-10%Y
22	15	35	100	10%	N	3#-1-10%N
23	15	35	200	10%	Y	3#-2-10%Y
24	15	35	200	10%	N	3#-2-10%N
25	15	35	100	20%	Y	3#-1-20%Y
26	15	35	100	20%	N	3#-1-20%N
27	15	35	200	20%	Y	3#-2-20%Y
28	15	35	200	20%	N	3#-2-20%N
29	15	35	100	40%	Y	3#-1-40%Y
30	15	35	100	40%	N	3#-1-40%N
31	15	35	200	40%	Y	3#-2-40%Y
32	15	35	200	40%	N	3#-2-40%N
33	15	35	100	60%	Y	3#-1-60%Y
34	15	35	100	60%	N	3#-1-60%N
35	15	35	200	60%	Y	3#-2-60%Y
36	15	35	200	60%	N	3#-2-60%N
37	15	35	100	80%	Y	3#-1-80%Y
38	15	35	100	80%	N	3#-1-80%N
39	15	35	200	80%	Y	3#-2-80%Y
40	15	35	200	80%	N	3#-2-80%N
41	15	35	100	100%	Y	3#-1-100%Y
42	15	35	100	100%	N	3#-1-100%N
43	15	35	200	100%	Y	3#-2-100%Y
44	15	35	200	100%	N	3#-2-100%N

Table S3. Elemental composition, elemental ratio and chemical bond concentration of ETFE membrane surface

Sample	Elemental composition (at%)			Elemental ratio (a.u.)		Contribution of main components of C1s (%)	
	C	F	O	F/C	O/C	290.8 eV	284.8 eV
						CH_2-*C*F_2	*C*-*C*/*C*-H
Untreated ETFE membrane	43.13	56.82	0.05	1.32	0	52.28	0
3#-2-0%N	49.35	42.03	8.62	0.85	0.17	39.47	12.05
3#-2-100%N	60.42	28.52	11.06	0.47	0.18	23.27	39.52
3#-2-100%Y	52.51	39.25	8.24	0.74	0.16	27.50	36.96
3#-1-60%N	54.79	34.86	10.35	0.63	0.18		
3#-2-60%N	63.53	24.89	11.58	0.39	0.18		
3#-1-60%Y	53.40	42.89	3.71	0.80	0.07		
3#-2-60%Y	57.53	34.43	8.24	0.59	0.14		

Reference

[1] S. Velasco, F.L. Roman, J.A. White, On the Clausius-Clapeyron Vapor Pressure Equation, JOURNAL OF CHEMICAL EDUCATION 86 (2009) 106-111. https://doi.org/10.1021/ed086p106

[2] S. Mohammadzadeh, G. Zahedi, A new vapor pressure equation for pure substances, Korean Journal of Chemical Engineering 25 (2008) 1514-1517.

[3] C. Smith, A. Barifcani, D. Pack, Helium substitution of natural gas hydrocarbons in the analysis of their hydrate, Journal of Natural Gas Science and Engineering 35 (2016) 1293-1300. https://doi.org/10.1016/j.jngse.2016.09.033
